# Acupuncture versus pharmacological conversation in treatment of atrial fibrillation in a randomized controlled trial: a systemic review and meta-analysis

**DOI:** 10.1186/s40001-022-00738-4

**Published:** 2022-07-04

**Authors:** Yibing Li, Jinming Song, Bangqi Wu, Xuhui Wang, Lin Han, Zhenzhen Han

**Affiliations:** 1 Tianjin University of Traditional Chinese Medicine, Tianjin, 301617 China; 2 National Clinical Research Center for Chinese Medicine Acupuncture and Moxibustion, First Teaching Hospital of Tianjin University of Traditional Chinese Medicine, Tianjin, 300381 China

## Abstract

**Objective:**

This study aimed to investigate the effect of conventional drugs combined with acupuncture therapy on the conversion of sinus rhythm in patients with atrial fibrillation.

**Methods:**

We searched databases, such as PubMed, Embase, WOS, Cochrane, CNKI (China National Knowledge Infrastructure), Wan fang Data, VIP, and CBM to collect data in randomized controlled trials of acupuncture included patients with atrial fibrillation. Publication time was limited from the beginning to May 15, 2021. The primary outcome is the number of participants who converted successfully.

**Results:**

A total of 11 papers were included in this study. The combined effect indicated that acupuncture significantly effectively benefitted the patients with atrial fibrillation (RR = 1.208, 95% CI 1.123, 1.298, *P* < 0.001). Further subgroup analysis of persistent and paroxysmal atrial fibrillation and the timing of acupuncture suggested that the addition of acupuncture was not statistically significant in the treatment of persistent AF compared to the control group (RR = 1.147, 95% CI 0.811, 1.623 *P* = 0.147). The combination of acupuncture was more effective in paroxysmal AF RR = 1.148 (95% CI 1.064, 1.239) *P* < 0.001. In addition, when the acupuncture time was limited to 20 min, it had the best treatment effect (RR = 1.510, 95% CI 1.25, 1.82).

**Conclusions:**

The combination of pharmacological resuscitation with acupuncture significantly improved the conversion of paroxysmal atrial fibrillation compared to pharmacological resuscitation only. The most significant benefit was achieved with an acupuncture duration of < 20 min. Thus, the combination of acupuncture could be considered in clinical practice for the resuscitation of patients with atrial fibrillation.

## Introduction

Atrial fibrillation (AF) is the most common clinical arrhythmia associated with atrial enlargement, myocardial damage, heart failure, and other organic heart diseases. In China, the prevalence of AF has been found to be approximately 0.77% [1] and is known to increase with age; it has also been reported to be more prevalent in men than in women of all age groups [2, 3]. In addition, AF has been shown to cause many other serious complications. Patients with AF are known to be at a 4–5 times higher risk of ischemic stroke compared to patients without AF, resulting in a mortality rate of approximately 20% and a disability rate of 60%. Asian patients with AF have a higher risk of ischemic stroke and hemorrhagic stroke than non-Asian patients [4]. In addition, AF triples the prevalence and exacerbates the symptoms of heart failure [5], increases the risk of myocardial infarction by twofold [6], and increases the risk of dementia, cognitive decline, and renal insufficiency [7, 8].

As an economical green therapy in traditional medicine, acupuncture has been used for the clinical treatment of AF [9, 10], and has been shown to safely and effectively relieve the symptoms of AF and reduce the recurrence rate after early RFCA (radiofrequency catheter ablation) and after electrical cardioversion. Acupuncture can also improve sleep and relieve anxiety and depression in patients with AF [11], improving their quality of life.

Although some studies have shown the effectiveness of acupuncture in the treatment of AF, these studies lack systematic analysis to verify the effectiveness and heterogeneity of acupuncture in AF. Here, we studied the effectiveness and safety of acupuncture for the treatment of AF as well as investigated the effects of different durations of acupuncture on the treatment efficacy.

## Research method

This study followed the international guidelines for reporting meta-analyses for the selection and use of research methods. This study protocol was registered with INPLASY (202150108).

### Literature inclusion and exclusion criteria

#### Study design

We used Randomized controlled trials (RCTs) and studies related to acupuncture for AF for this meta-analysis.

#### Inclusion criteria of the literature

(1) The study population included patients with AF, and the diagnostic criteria for AF followed the European Society of Cardiology Guidelines for the Management of AF 2020 [12]; (2) patients who had been treated with acupuncture; (3) the type of study was a randomized controlled trial.

#### Exclusion criteria of the literature

(1) The studies that were conducted in patients without AF; (2) the studies without a comfort group; (3) the article was repeatedly published.

### Literature search strategy

Eight databases, including PubMed, web of science, Cochrane, Embase, China Biomedical Literature System, CNKI, VIP database, and Wan fang database, were searched for RCTs based on acupuncture for AF. The search period ran from the start date of each database to May 16, 2021. The search strategy was based on the principles of PICOS (overall, intervention, comparison, outcome, and study design), and was done using a combination of subject terms and free words, identified by repeated pre-searching, and supplemented by manual searches and reference tracking. Chinese search terms included atrial fibrillation, paroxysmal atrial fibrillation, persistent atrial fibrillation, acupuncture, electroacupuncture, acupuncture points, meridians, clinical studies, clinical trials, controlled clinical trials, randomized controlled trials, and pragmatic clinical trials. The English search terms for WOS were as follows:

#1 TS = (Acupuncture Treatment or Acupuncture Treatments or Treatment, Acupuncture or Therapy, Acupuncture or Pharmacoacupuncture Treatment or Treatment, Pharmacoacupuncture or Pharmacoacupuncture Therapy or Therapy, Pharmacoacupuncture or Acupotomy or Acupotomies or acupuncture or Needle or Needling or Electroacupuncture).

#2 TS = (Atrial Fibrillations or Fibrillation, Atrial or Fibrillations, Atrial or Auricular Fibrillation or Auricular Fibrillations or Fibrillation, Auricular or Fibrillations, Auricular or Persistent Atrial Fibrillation or Atrial Fibrillation, Persistent or Atrial Fibrillations, Persistent or Fibrillation, Persistent Atrial or Fibrillations, Atrial Fibrillation, Familial or Atrial Fibrillations, Familial or Familial Atrial Fibrillations or Fibrillation, Familial Atrial or Fibrillations, Familial Atrial or Paroxysmal Atrial Fibrillation or Atrial Fibrillation, Paroxysmal or Atrial Fibrillations, Paroxysmal or Fibrillation, Paroxysmal Atrial or Fibrillations, Paroxysmal Atrial or Paroxysmal Atrial Fibrillations or AF).

#3 TS = (Clinical Study or Clinical Trial or Controlled Clinical Trial or Randomized Controlled Trial) #4 #1 AND #2 AND #3.

#### Interventions

The test group received acupuncture plus pharmacological resuscitation, while the control group received pharmacological resuscitation alone.

#### Outcome indicators

Patients with AF after surgery were observed to determine the rate of AF recurrence. Patients without surgery were studied to observe the rate of conversion of paroxysmal AF or persistent AF to sinus rhythm after acupuncture.

### Literature screening, data extraction, and quality assessment

#### Literature search

Two investigators screened the literature and extracted relevant data using an independent double-blind method based on the inclusion and exclusion criteria. If there is disagreement at the mutual review, screening, and data extraction stages, a third researcher joined the discussion on whether to include these data.

#### Data extraction

Data extracted from the literature included mainly author names, year of publication, sample size, age, gender, and time of needling. The results extracted from the study were indicators of outcomes included in the literature. Based on the extracted data, the needling time was categorized as < 20 min, 20–30 min, and > 30 min (Table 1).

**Table 1:** Basic information of the included clinical studies

Study	Junkui Yin	Albert Lomuscio	Baozhen Xu	Ying Jiao	Baode Han	Yuanzeng Zhang	Yuanshi Xia	Yahong Yan	Li Chen	Hongke Xu	Xuhai Wang
Age (mean ± SD)	61.6 ± 10.6	–	63 ± 7	–	64	–	–	52.1 ± 6.7	62.3 ± 5.4	58.9 ± 10.5	–
Gender (male:female)	53:32	–	58:50	243:189	64:50	–	–	34:26	32:28	52:28	–
Classification	Persistent	Persistent	Paroxysm	Paroxysm	Paroxysm	Paroxysm	Paroxysm	Paroxysm	Paroxysm	Paroxysm	Paroxysm
	AF	AF	AF	AF	AF	AF	AF	AF	AF	AF	AF
Duration of treatment	20 min	15–20 min	30 min	24 h	30 min	20 min	Pull out after the treatment	30 min	24 h	1 h	–
Intervention	Needle pricking & Amiodarone	Needle pricking	Needle pricking & Wenxin Granules	Needle pricking	Needle pricking & Digilanid C	Needle pricking	Needle pricking & Digilanid C	Needle pricking & Cardiox	Needle pricking & Amiodarone	Needle pricking	Needle pricking
Participants	85	80	108	432	114	71	90	60	60	80	54
Study design	Randomized; parallel-group clinical study	Randomized; parallel-group clinical study	Randomized; parallel-group clinical study	Randomized; parallel-group clinical study	Randomized; parallel-group clinical study	Randomized; parallel-group clinical study	Randomized; parallel-group clinical study	Randomized; parallel-group clinical study	Randomized; parallel-group clinical study	Randomized; parallel-group clinical study	Randomized; parallel-group clinical study
Study location	China	South Korea	China	China	China	China	China	China	China	China	China
Year	2019	2011	2015	1997	2012	2003	2014	2014	2012	2007	2007

#### Quality assessment

The risk of bias criteria was based on the Cochrane Collaboration Network's RCT criteria to qualitatively evaluate seven aspects of random sequence generation, distribution concealment, subject and investigator blinding, blinding of outcome assessors, incomplete outcome data, and selective reporting, and were evaluated as ‘‘low risk of bias,’’ ‘‘uncertain risk of bias,’’ and’’ (Table 2).

**Table 2:** Assessment of bias included all studies

Study	Year	Random sequence generation	Allocation concealment	Blinding of participants and personal	Blinding of outcome assessment	Incomplete outcome data	Selective reporting	Other bias
Junkui Yin	2019	L	L	H	H	L	L	U
Özlem Ceyhan	2020	L	L	H	H	L	L	U
ALBERTO LOMUSCIO	2011	L	L	H	H	L	L	U
Baozhen XU	2015	L	L	H	H	L	L	U
Ying Jiao	1997	L	L	H	H	L	L	U
Baode Han	2012	L	L	H	H	L	L	U
Yuanzeng Zhang	2003	L	L	H	H	L	L	U
Yuanshi Xia	2014	L	L	H	H	L	L	U
Yahong Yan	2014	L	L	H	H	L	L	U
Li Chen	2012	L	L	H	H	L	L	U
Hongke Xu	2007	L	L	H	H	L	L	U

#### Statistical analysis

The literature data were processed using Stata (v15.1); combined effect sizes and heterogeneity tests were performed, and forest plots were drawn. The literature outcome indicators were dichotomous variables, and the effect size was chosen as relative risk (RR) with an effect size of 95% of the confidence interval (95% CI). The meta-analysis followed strict PRISMA guidelines and tests of heterogeneity were performed using *P* values and *I*^2^. If there was no statistical heterogeneity between the results of these studies (*I*^2^ ≤ 40%, *P* > 0.1), a fixed-effects model was selected (dichotomous variables using the M–H method). If there was statistical heterogeneity between studies, the source of heterogeneity was further explored using meta-regression or subgroup analysis, and if the source of heterogeneity was unclear, a random-effects model (D + L method) was used for analysis.

## Results

**Fig. 1:**
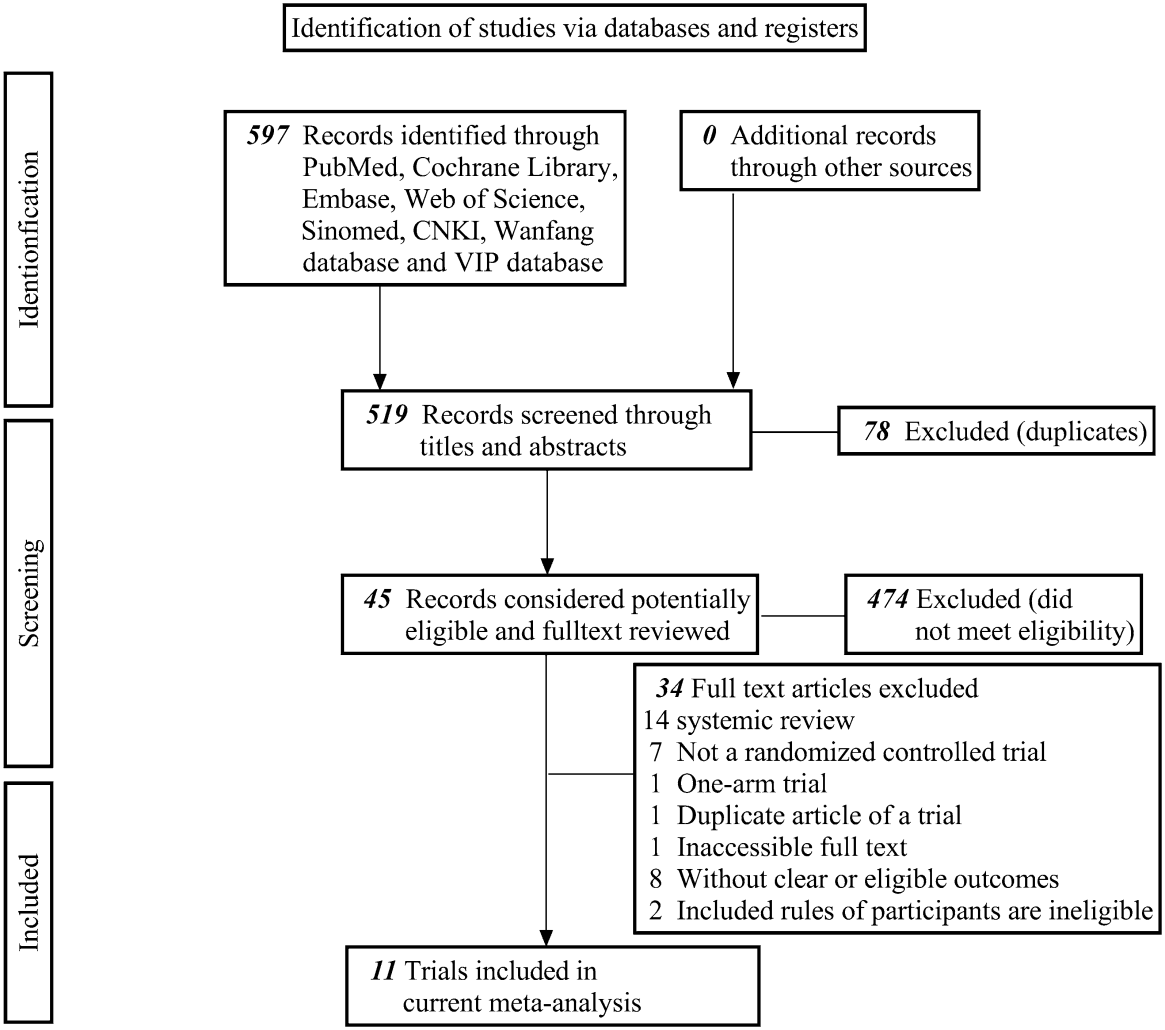
Flow chart for selected literature

A total of 597 articles was retrieved from the database, of which 78 duplicate papers were excluded. After browsing through the titles and abstracts, 474 papers were excluded. After reading the full text, 34 articles were excluded, of which 14 were reviews, 7 were non-RCTs, 1 was a single-arm trial, 2 included patients with sinus bradycardia after modified maze surgery for AF, 8 had unclear outcome indicators or did not fit the current study, 1 was a duplicate publication, and 1 could not be accessed in full text. We identified 11 potentially eligible trials. We included 11 trials of acupuncture for AF (1234 patients) [13–23] (Fig. 1).

**Fig. 2:**
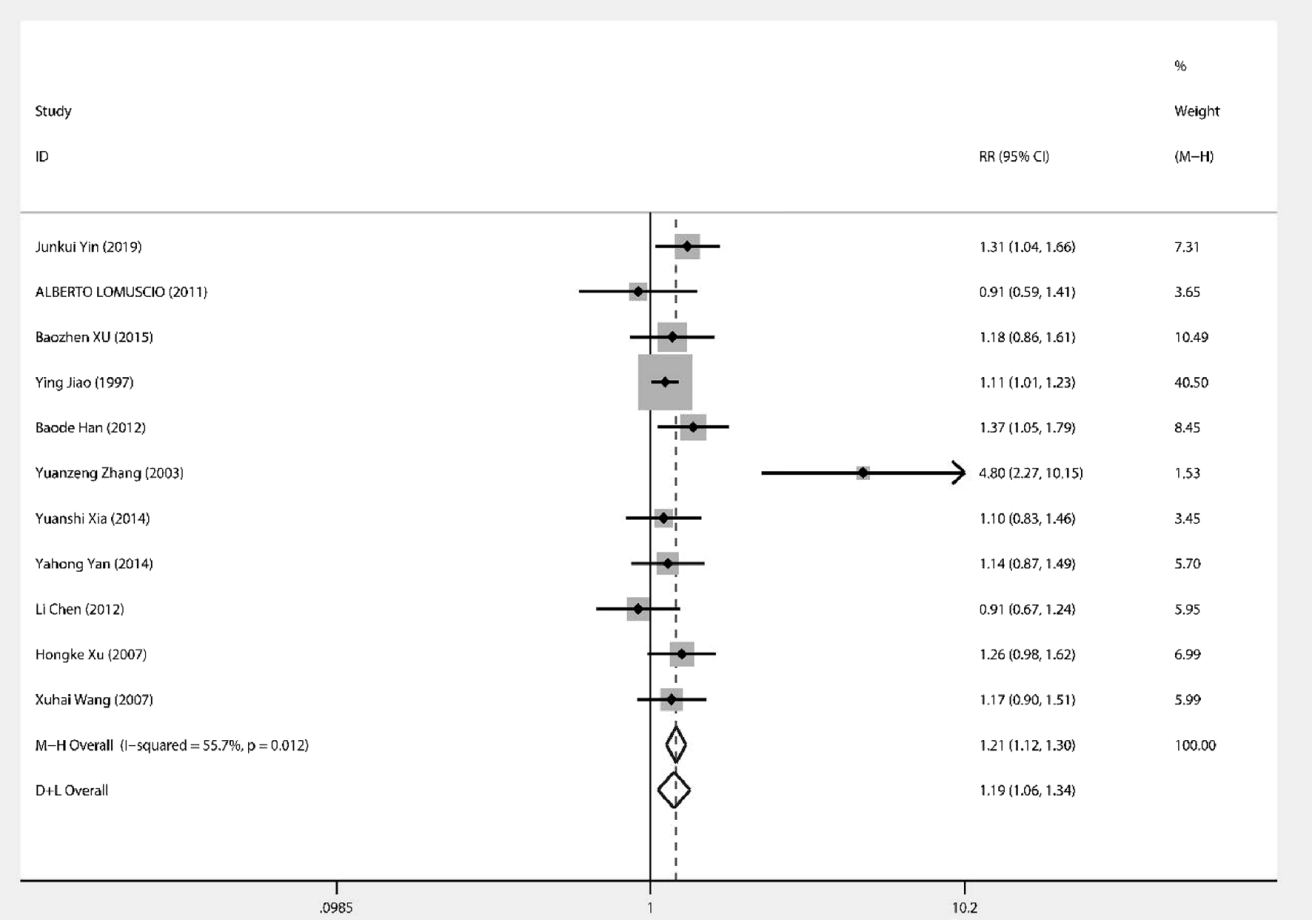
Forest plot for all studies

**Fig. 3:**
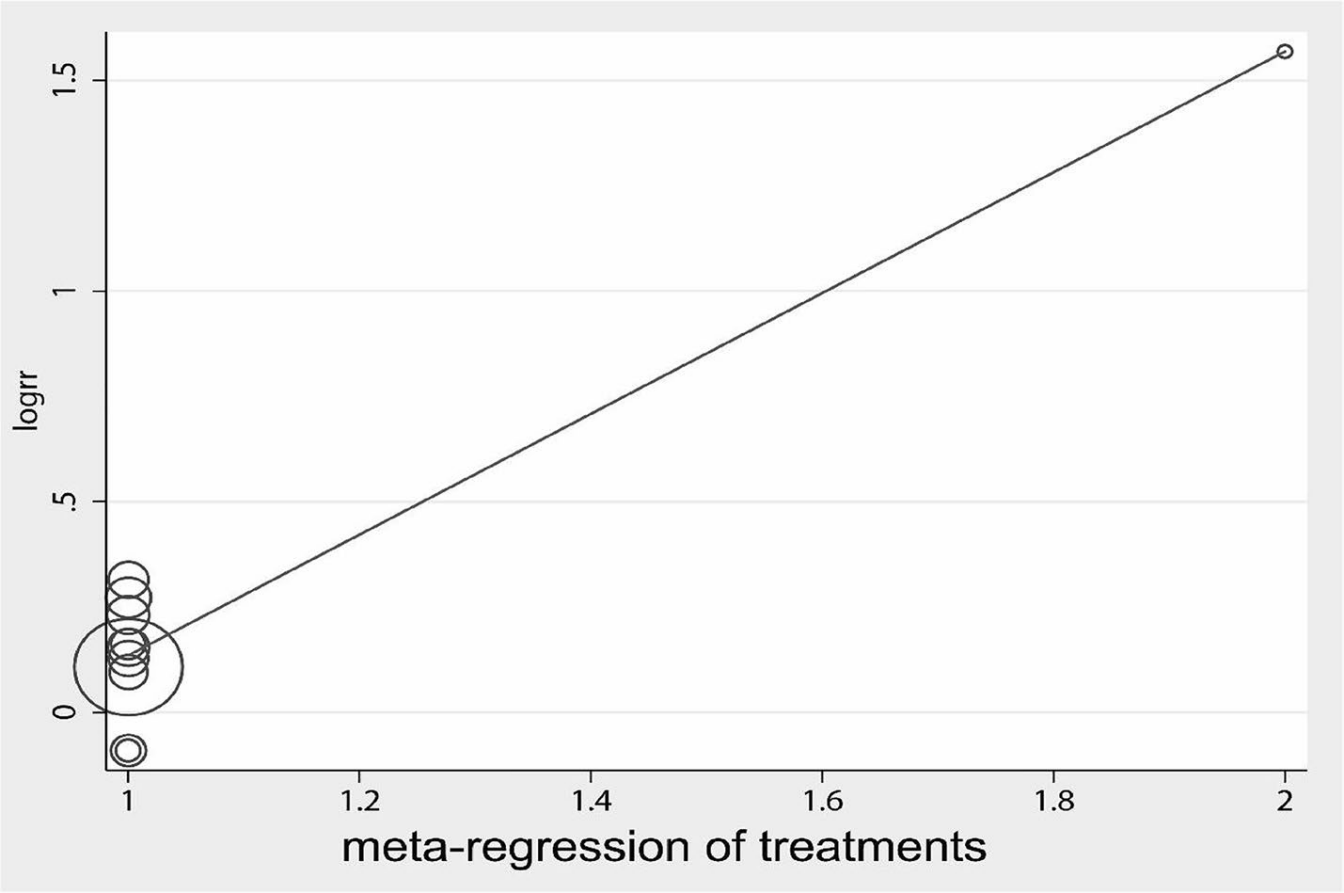
Meta-regression between different acupuncture points

**Fig. 4:**
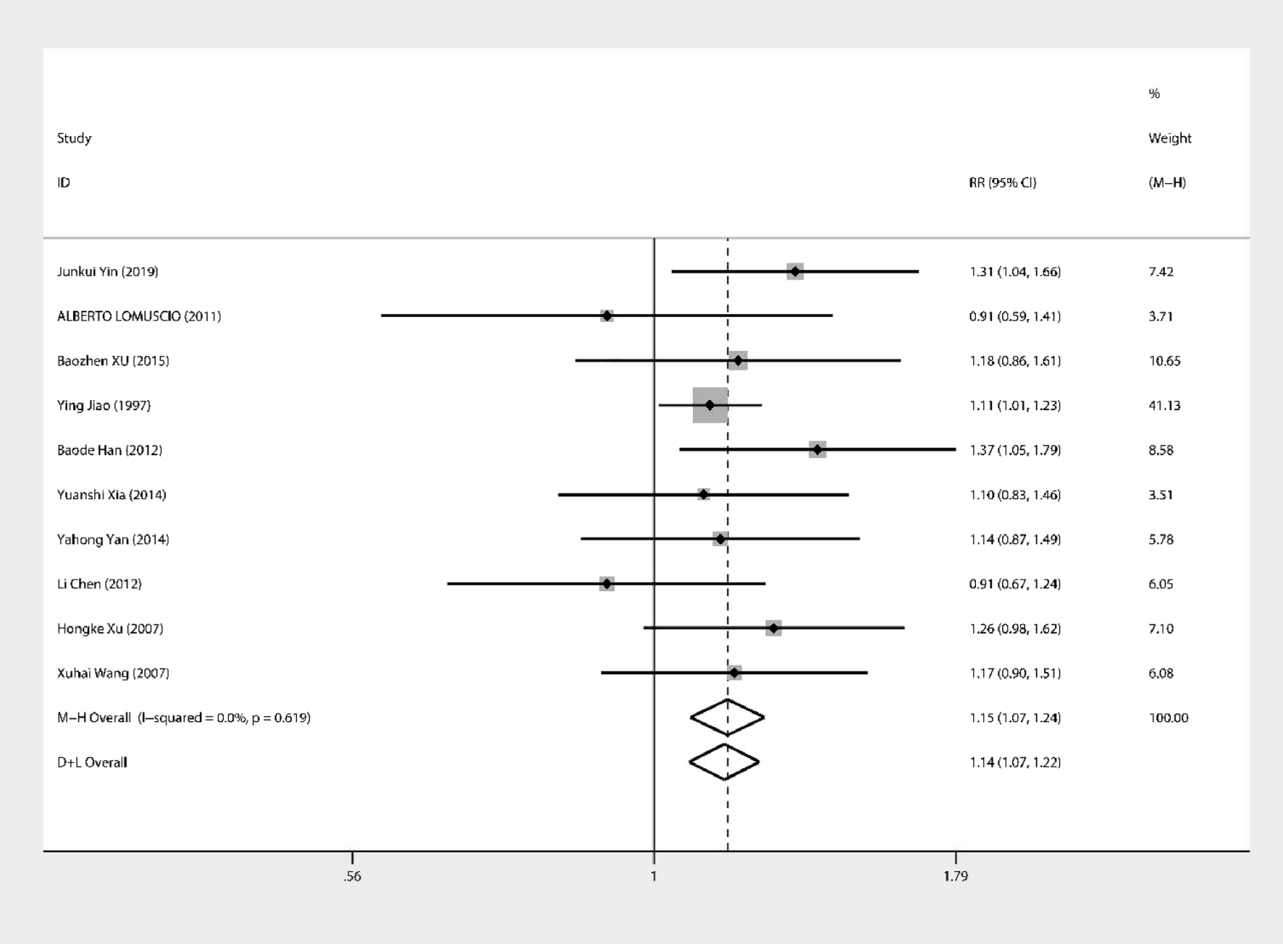
Forest plot included a similar method in needle pricking

Eleven papers investigated the therapeutic effects of acupuncture on the resetting of AF. The combined effect size RR = 1.19 (95% CI 1.06, 1.34) and heterogeneity analysis showed *I*^*2*^ = 55.7%, *P* = 0.01 (Fig. 2). Therefore, further searching for heterogeneity, regression analysis showed (Fig. 3) that heterogeneity was caused by differences in acupuncture point coefficient = − 1.30, standard error = 0.39, *P* < 0.001, 95% CI (− 2.18, − 0.42), excluding the following literature for analysis, *I*^2^ = 0.0, *P* = 0.619, no significant heterogeneity. The combined effect size was 1.21, 95% CI (1.123, 1.298), *P* < 0.001, which showed a significant improvement in the test group with the addition of acupuncture treatment (Fig. 4).

**Fig. 5:**
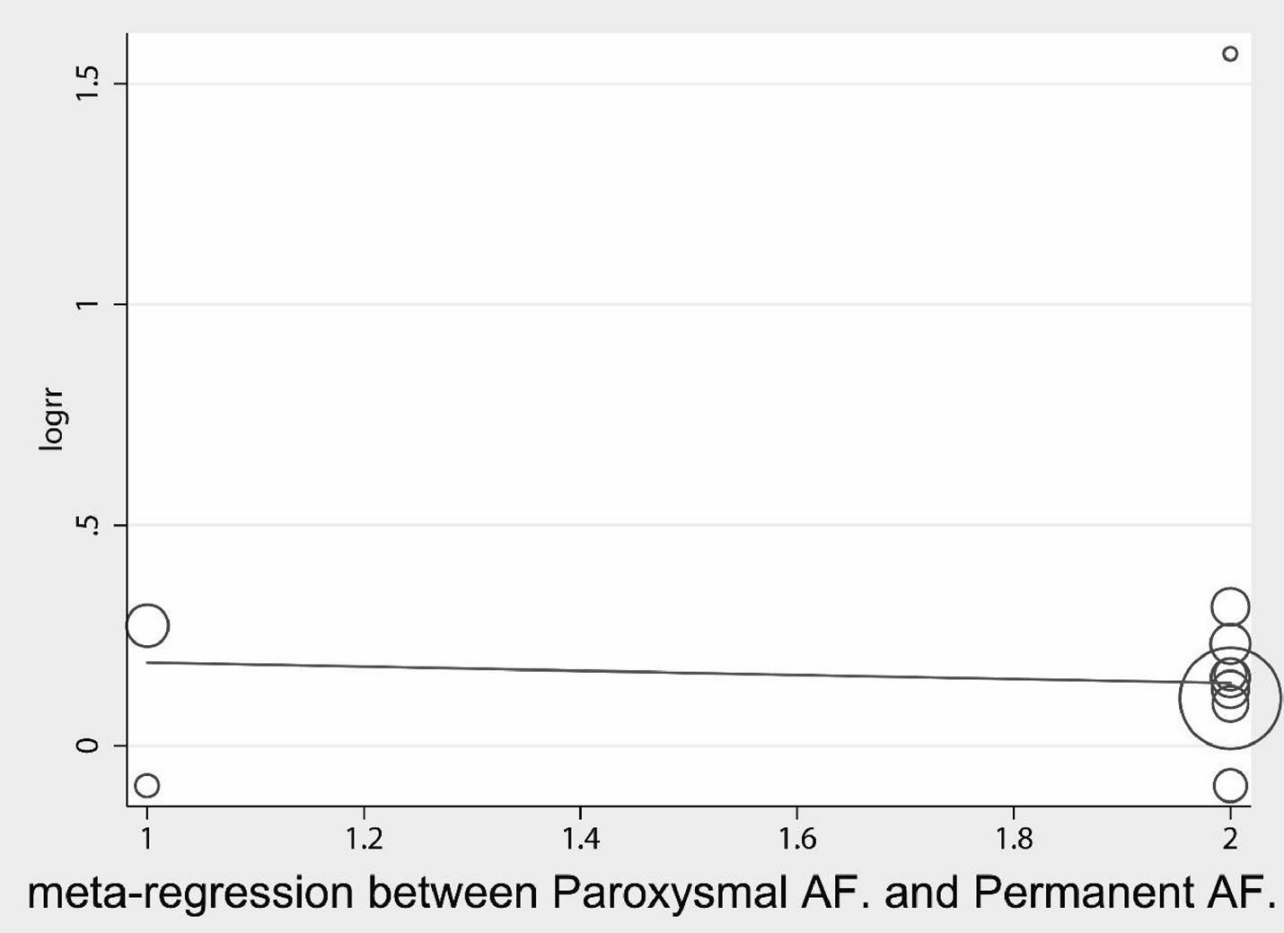
Meta-regression for both Paroxysmal AF and Persistent AF

**Fig. 6:**
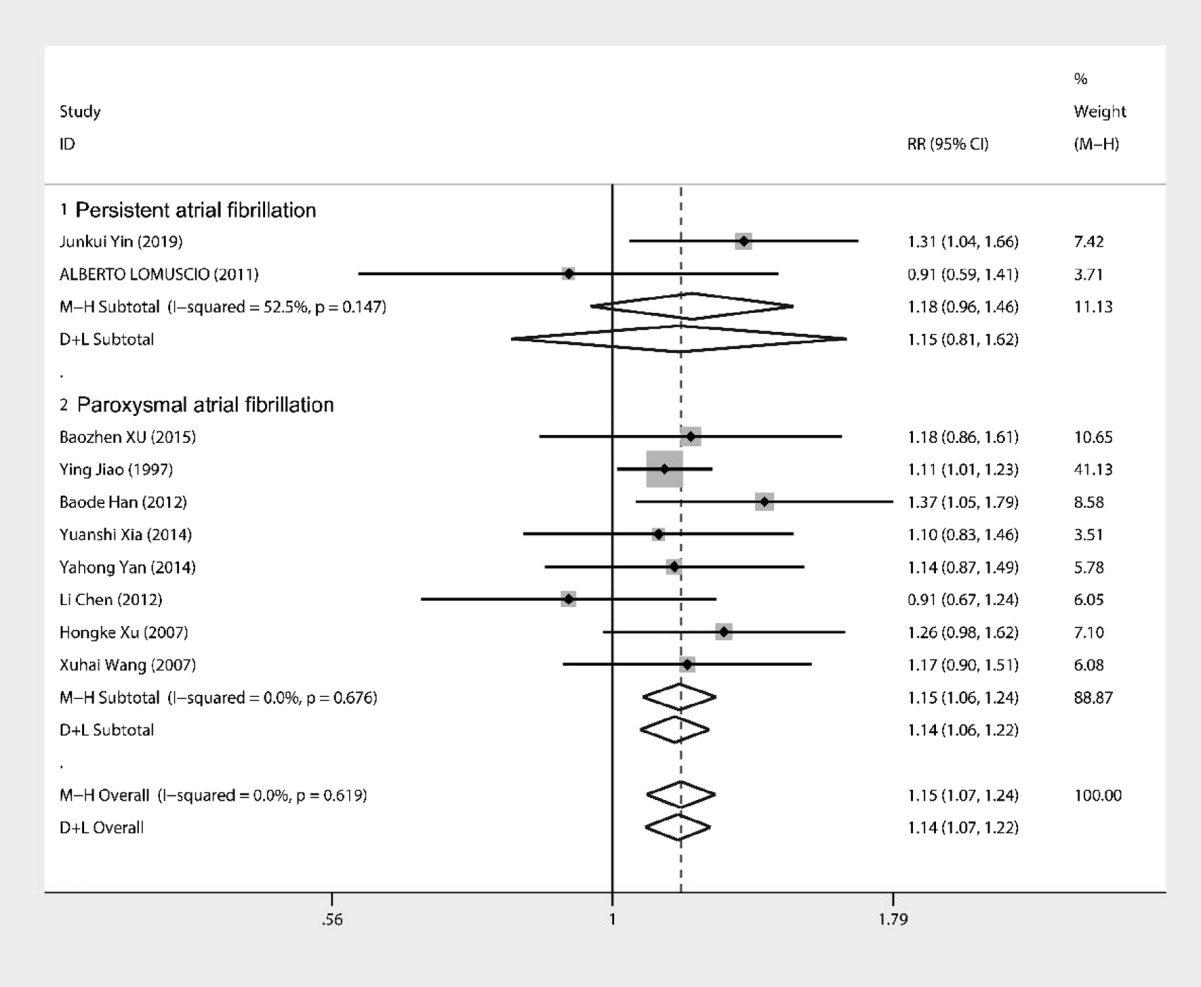
Subgroup analysis for dividing into Paroxysmal AF and Persistent AF

Further subgroup and regression analyses were performed for other factors. Regression analysis of disease type into persistent and paroxysmal AF showed no statistically significant correlation (Fig. 5) (coefficient = 0.24, SD = 0.33, *P* = 0.492; 95% CI − 0.51, 0.98). Subgroup analysis (Fig. 6) showed persistent AF group *I*^2^ = 52.5%, *P* = 0.15, combined effect size using the D + L method, showing an RR = 1.147 (95% CI 0.81, 1.62) *P* = 0.15. Paroxysmal AF *I*^2^ = 0.0%, *P* = 0.68, combined effect size using the M–H method, showing an RR = 1.15 (95% CI 1.064, 1.239) *P* < 0.001. These results showed that for paroxysmal AF, the addition of acupuncture was more effective, with a statistically significant difference between the test and the control groups, and a less significant effect for persistent AF, possibly related to the number of studies.

**Fig. 7:**
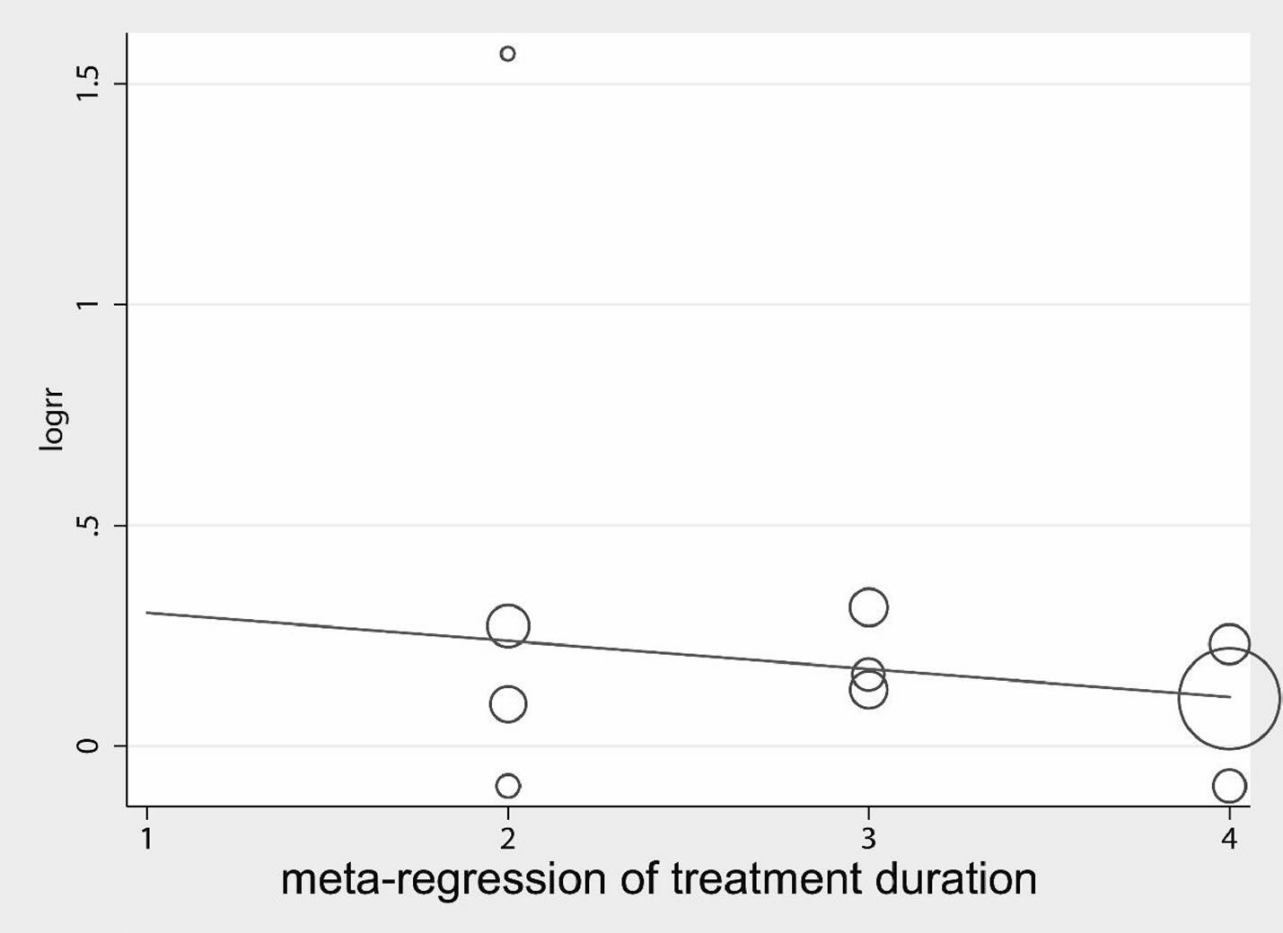
Meta-regression for different treatment duration (within 20 min; 20–30 min; over 30 min)

**Fig. 8:**
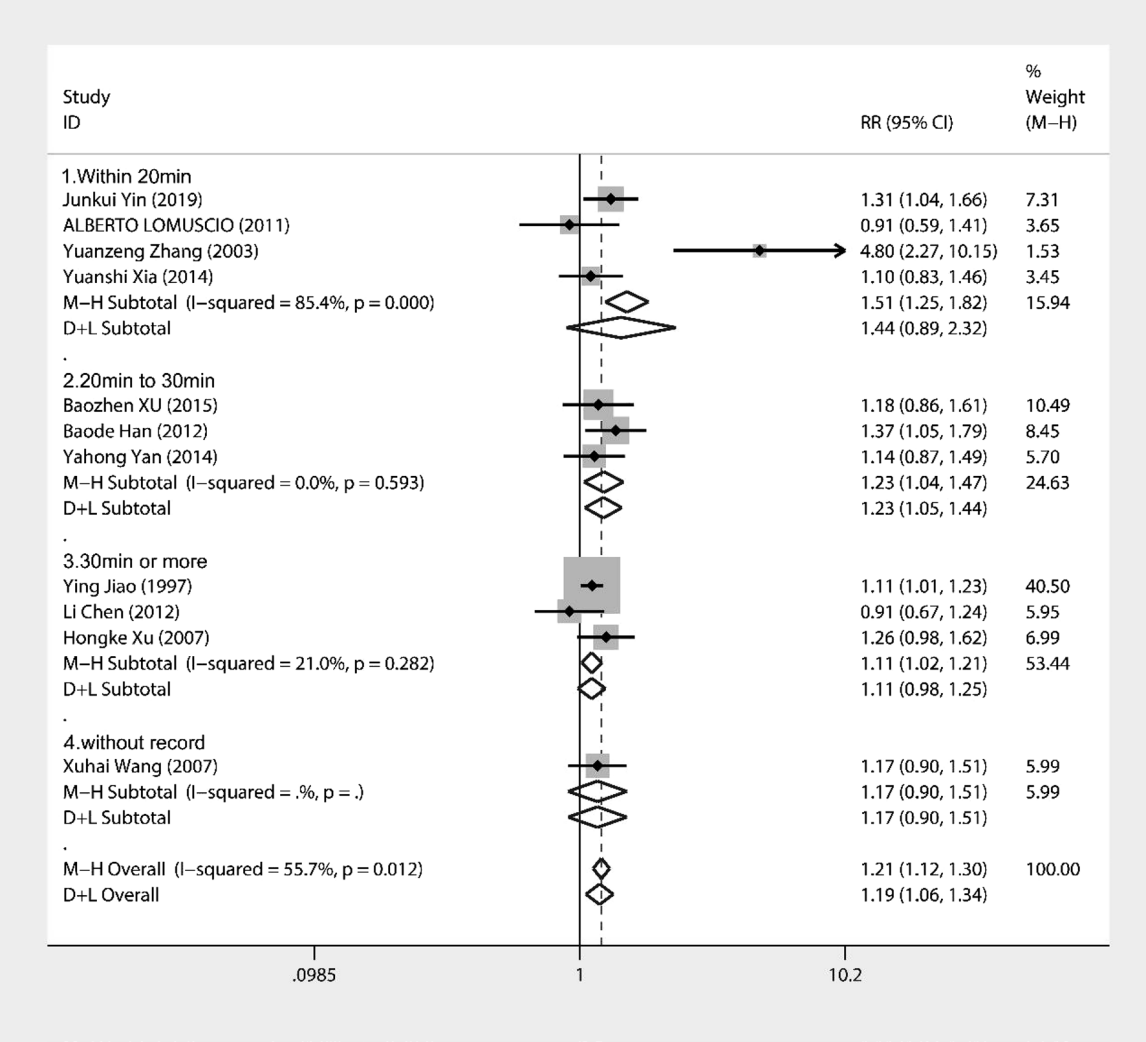
Forest plot for different treatment durations (within 20 min; 20–30 min; > 30 min)

Regression analysis (Fig. 7) was performed by dividing the duration of needling into four groups: (1) within 20 min, (2) 20–30 min, and (3) > 30 min. The results showed a coefficient = 0.26, Std. Err. = 0.16, *P* = 0.16 (95% CI − 0.12, 0.63) It was possible that there was no significant positive association between the duration of needling and the effect of treating AF. However, subgroup analysis (Fig. 8) showed that the best RR = 1.44 (95% CI 0.89, 2.32) was within 20 min of needling time, the next best RR = 1.23 (95% CI 1.04, 1.47) was between 20 and 30 min and the worst RR = 1.11 (95% CI 1.02, 1.21) was > 30 min. Considering that the duration of treatment for persistent AF was all within 20 min, it was presumed that needling within 20 min was more effective for the treatment of AF.

**Fig. 9:**
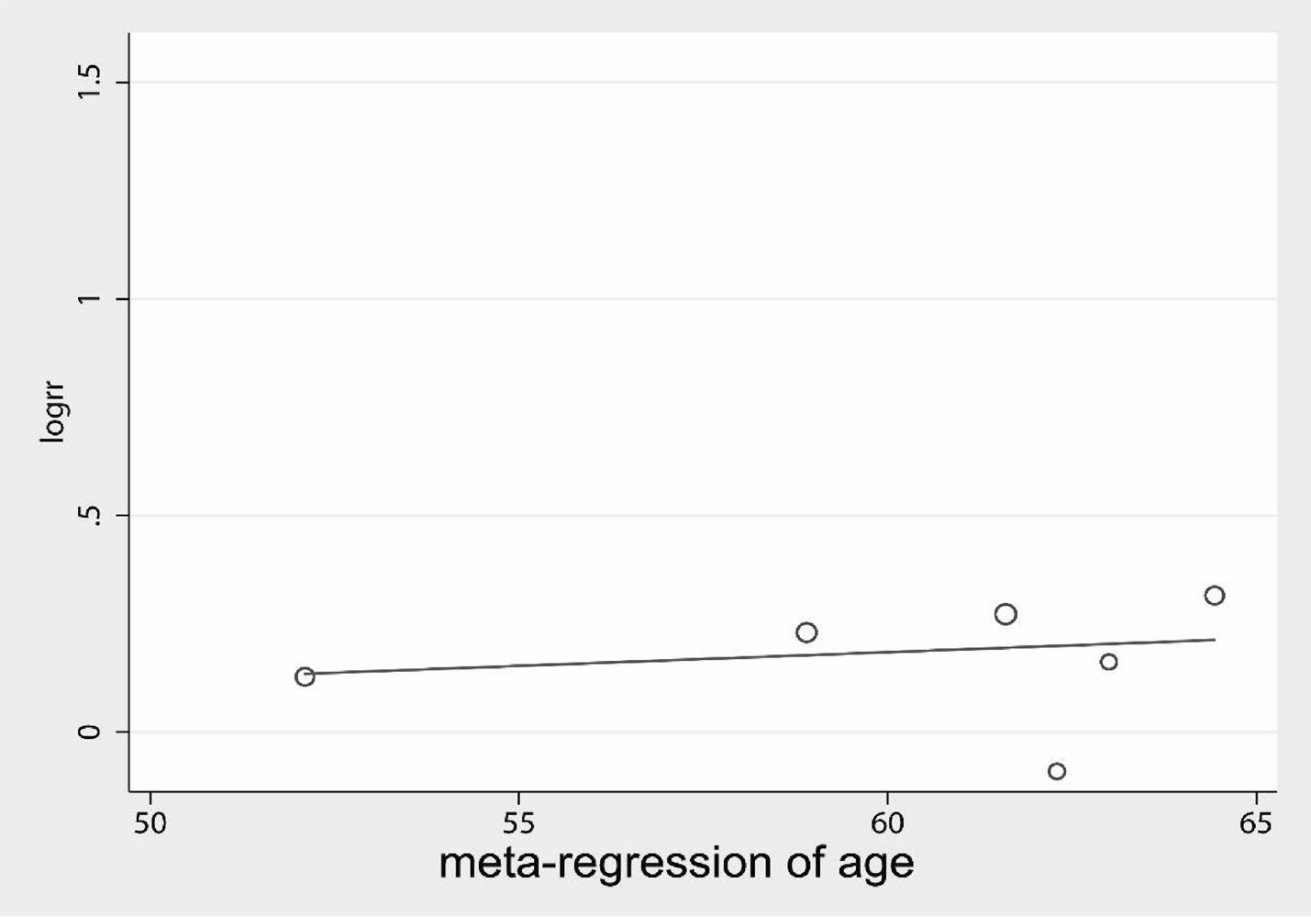
Meta-regression for mean age

**Fig. 10:**
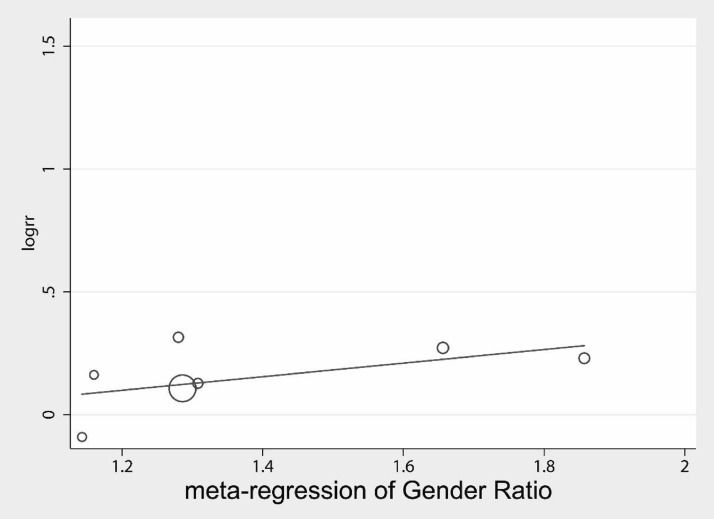
Meta-regression for gender ratio

Further the confounding factors, such as age and sex were included in the regression analysis; the age regression (Fig. 9) showed that age was not associated with treatment effect, Coef. = − 0.19, Std. Err. = 0.91, *P* = 0.841 (95% CI − 2.70, 2.32). The sex ratio regression (Fig. 10) showed no significant association between sex and treatment effect, Coef. = − 0.23, Std. Err. = 0.26, *P* = 0.42 (95% CI − 0.91, 0.44).

### Bias test

**Fig. 11:**
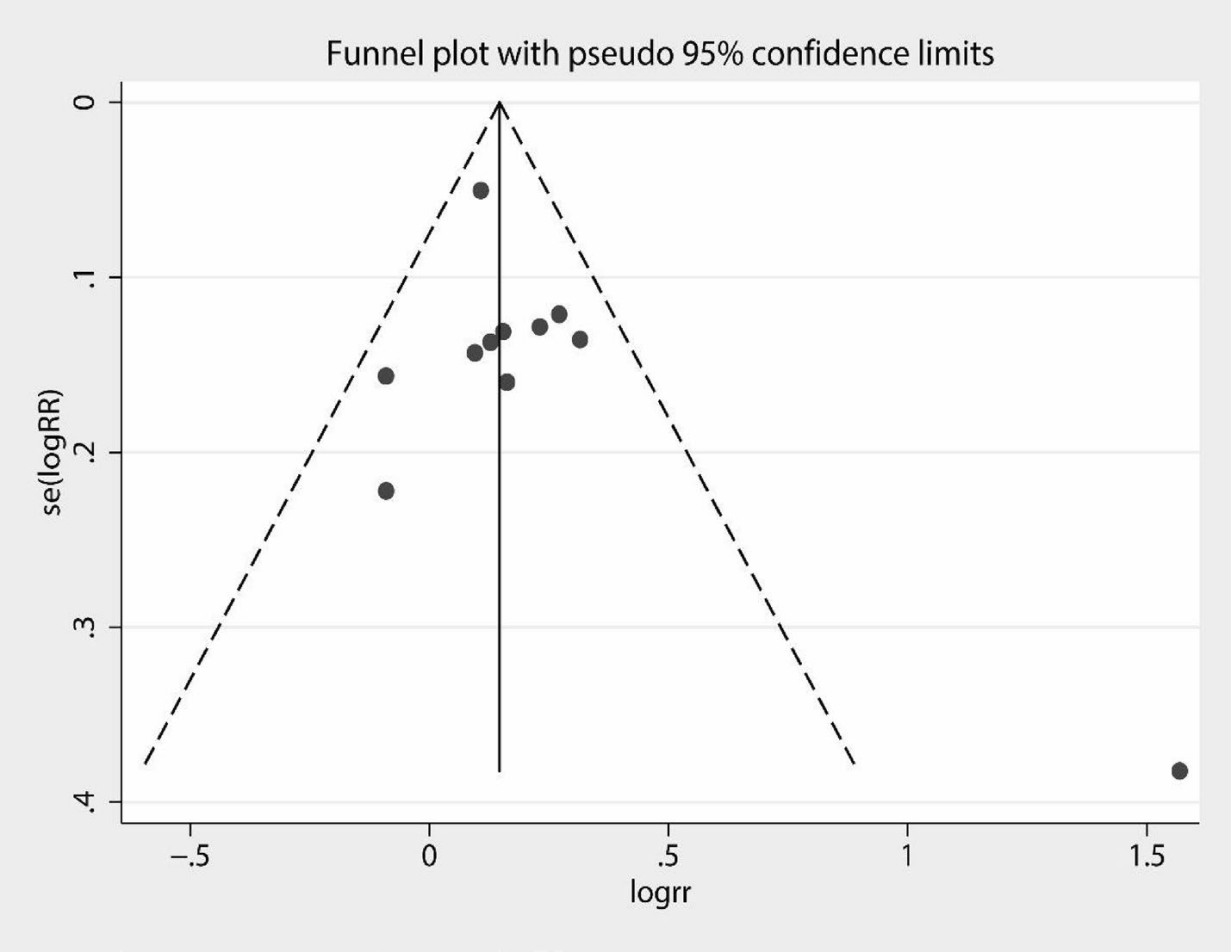
Funnel plot for evaluating bias

**Fig. 12:**
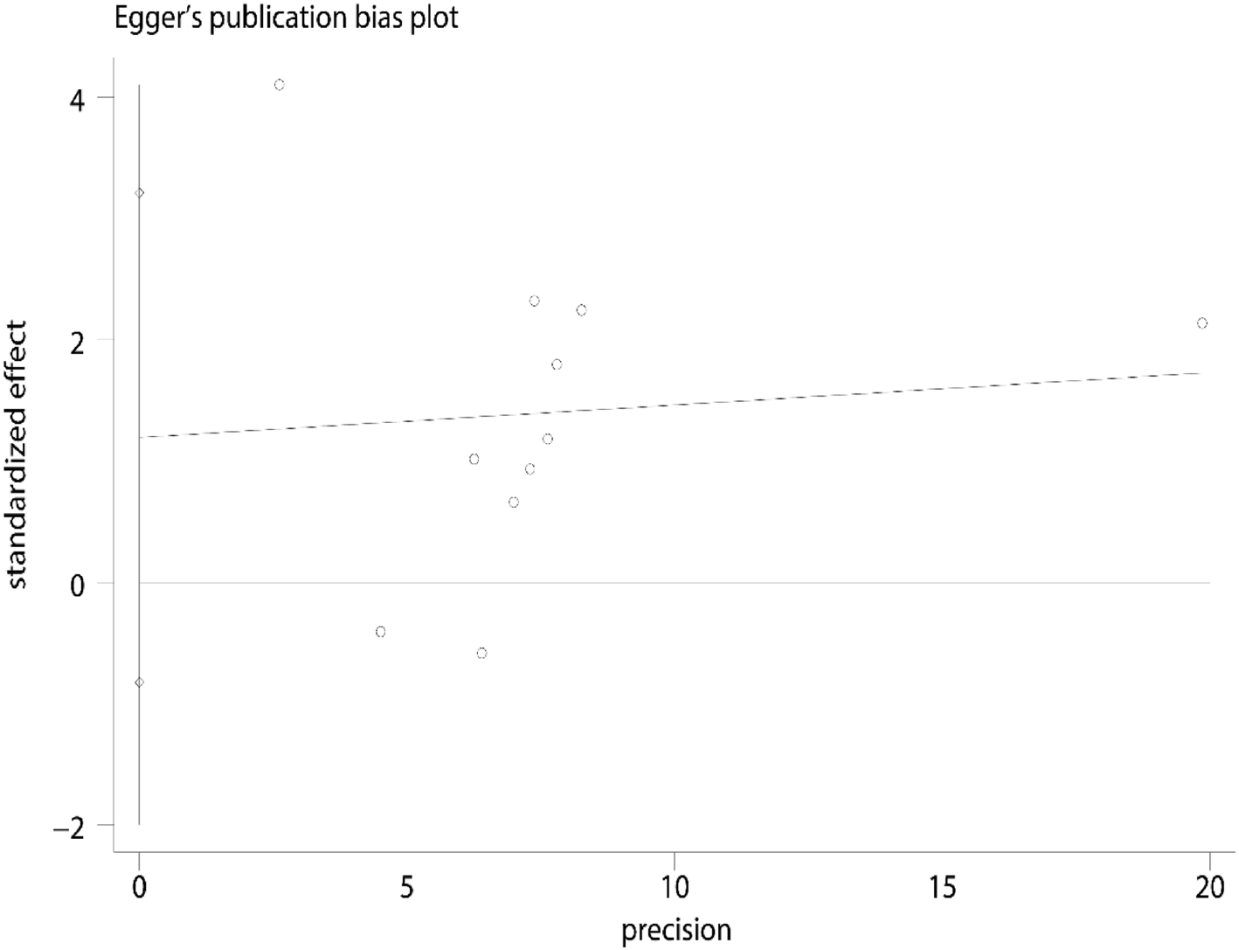
Egger's test

**Fig. 13:**
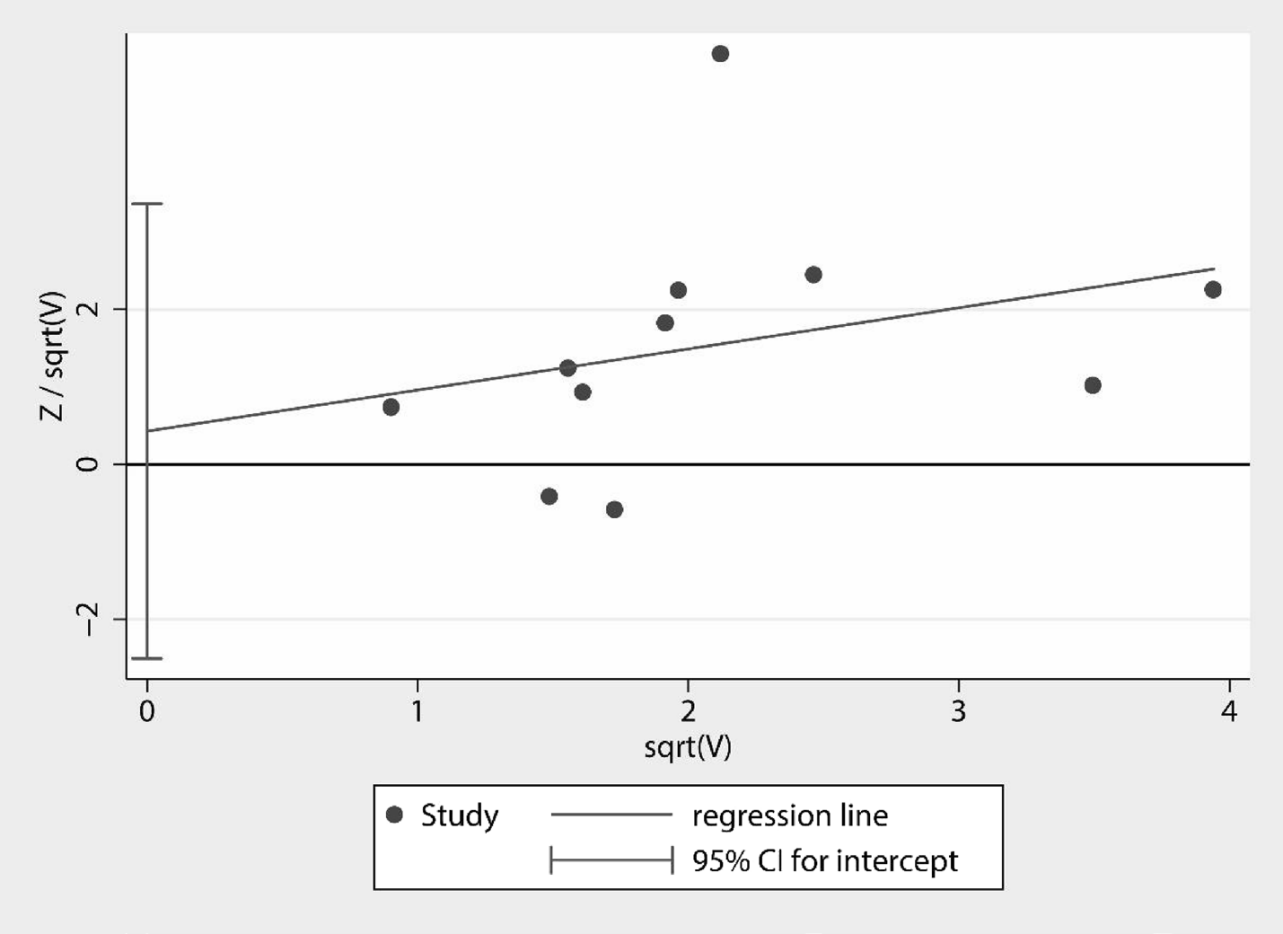
Harbord's modified test

Visual observation of the funnel plot (Fig. 11) and the possible bias egger's test (Fig. 12) showed *P* = 0.239 (95% CI − 0.99, 3.42). Harbord's modified test (Fig. 13) showed *P* = 0.748 (95% CI − 2.51, 3.37), suggesting no significant publication bias.

## Discussion

The results of this study demonstrated that acupuncture could restore sinus rhythm and improve the ventricular rate and rhythm control in patients with AF. The results of this study were consistent with previous studies and reaffirmed the effectiveness of acupuncture in the treatment of AF.

The pathophysiology of AF revolves around the promotion of ectopic discharge and folding mechanisms in four main areas: ion channel dysfunction, abnormal Ca^2+^ handling, structural remodeling, and impaired autonomic regulation [24]. It was found [25] that acupuncture could reduce the expression of L-type Ca^2+^ channels and Cl-channels and stop excessive Ca^2+^ inward flow. Acupuncture at the Neiguan point could reduce Cx40 expression in atrial tissue [26], reduce the degree of damage to atrial myofibers and mitochondria, reduce damage to atrial myocyte structures [27, 28], and delay the process of atrial structural remodeling. Acupuncture could also regulate autonomic balance, with vagal excitability increasing under acupuncture stimulation and vagal excitation decreasing, and sympathetic excitation increasing after stimulation disappears [29–31].

Nine studies compared the effects of acupuncture and drugs on atrial fibrillation, and found that the acupuncture group was significantly better than the drug group in converting atrial fibrillation to sinus rhythm. However, none of the above studies conducted follow-up to explore the recurrence rate of tremor and the maintenance time of acupuncture effect on cured patients. It was very necessary to effectively control the recurrence of atrial fibrillation for patients who had converted sinus rhythm. Two studies compared the effects of acupuncture on the recurrence of atrial fibrillation in patients after electrical cardioversion or radiofrequency ablation. It was found that the recurrence rate of atrial fibrillation in the acupuncture group was significantly lower than that in the control group, suggesting that acupuncture could effectively reduce the recurrence rate of atrial fibrillation for patients who have converted to sinus rhythm. The subjects in these two studies were all converted to sinus rhythm after electrical cardioversion or radiofrequency ablation. There was currently no such study on the recurrence rate of atrial fibrillation in patients who converted to sinus rhythm with drugs or only using acupuncture. Therefore, the maintenance effect of acupuncture in the treatment of atrial fibrillation cannot be evaluated.

This was probably related to the fact that the Yingxiang acupuncture point regulated the excitability of the sensory nerves, parasympathetic nerves, and sympathetic nerves in the nasal mucosa, as well as the release of the corresponding neuropeptides and the reduction of inflammatory factors [32, 33]; however, since only one paper used the Yingxiang acupuncture point, it was not significant enough to suggest that the specificity of the Yingxiang acupuncture point was higher than that of other acupuncture points. The other trials included in this study (except for the Yingxiang acupoint and the acupuncture point) used the Neiguan acupoint. This was probably related to the acupoint specificity of the Neiguan point, which was found to affect the firing frequency of the amygdala and regulate autonomic balance [34], as well as to reduce the expression of c-fos cells in the nucleus tractus solitarius, thereby counteracting the activation of neurons in the nucleus tractus solitarius by afferent information brought on by arrhythmias and reducing heart rate [35]. Acupuncture at the Neiguan point also reduced the levels of the inflammatory factors, such as CRP, IL-8, and TNF-α and improved the index of heart rate variability [36], which were closely associated with the development and persistence of AF [13, 37].

In this study, we found that needle retention time < 20 min was the most effective for AF, 20–30 min was the second most effective, and > 30 min had the lowest benefit. According to Lin [38], similar to the metabolic process of drugs in the body, the duration of acupuncture could be divided into the optimal induction period, half-life, and residual effect period. For AF, the optimal induction period could be reached rapidly after acupuncture with maximum acupuncture effect, but too long a time might produce acupuncture tolerance and acupoint fatigue resulting in poor results. Therefore, the best efficacy was achieved at acupuncture times of < 20 min, while effects at > 30 min were rather poor. However, the sample sizes of the included studies are generally small, and there are not enough studies on acupuncture for AF to draw definitive conclusions.

Patients with AF are usually treated with anticoagulants, such as warfarin or newer anticoagulants (NOACs), and some doctors and patients wonder whether needling might increase the risk of bleeding. A meta-analysis [39] of 428 patients showed that needling after anticoagulants and antiplatelet agents did not increase the incidence of bleeding-related adverse events. A meta-analysis [40] including 316 patients showed that acupuncture was safe for patients with AF taking NOAC and did not increase the risk of bleeding. Another meta-analysis [41] that included 384 patients also showed that acupuncture was safe in anticoagulated patients, provided that the location and depth of acupuncture were assured. These studies showed that acupuncture was safe in the treatment of patients with AF who were taking anticoagulants without an increased risk of bleeding and associated adverse events when the acupuncturist was trained in a standardized manner.

In the early stage of the application of acupuncture to atrial fibrillation, the effect of acupuncture alone in converting to sinus rhythm in patients with atrial fibrillation was better than antiarrhythmic drugs. With the development of modern medicine and the improvement of clinical guidelines, the anti-arrhythmic advantages of acupuncture were no longer, but the extracardiac side effects of anti-arrhythmic drugs were greater. Acupuncture combined with anti-arrhythmic drugs reduced the incidence of adverse events and enhanced the treatment effect. Catheter ablation therapy was superior to antiarrhythmic drugs in maintaining sinus rhythm and improving quality of life [42, 43], but its postoperative rate of recurrence was high, and new-onset atrial tachycardia with antiarrhythmic drugs might occur during the 3 month postoperative gap. Catheter ablation was efficacious in the treatment of atrial fibrillation, but it might be ineffective in preventing recurrence of atrial fibrillation at 6 months, even if oral antiarrhythmic drugs were adhered to after the procedure [44]. Since surgery was expensive and patients had a heavy medical burden, and acupuncture could reduce their postoperative atrial fibrillation recurrence rate, the difference in efficacy of different acupuncture treatment amounts to prevent recurrence rate after radiofrequency ablation in patients with atrial fibrillation may be a future research direction. It is also expected that future studies will explore the mechanism of action of acupuncture after catheter ablation from multiple perspectives and establish evidence-based evidence of acupuncture in the treatment of atrial fibrillation.

## Limitations

This study had several limitations. (1) The methodology of some of the included papers was not comprehensive, and some of the biases could not be ruled out. (2) The heterogeneity of persistent AF was large, the number of papers was small, and the results of the random-effects model were not reliable. (3) The acupuncture points were not clearly described. As a result, the study results could be biased to some extent. Thus, further experimental studies are needed to support the efficacy of acupuncture in AF.

## Conclusions

These studies found that the combination of acupuncture was significantly more effective in the treatment of AF compared to conventional pharmacological resuscitation, with the greatest benefit being achieved with acupuncture times of < 20 min. However, larger, more rigorous RCTs are needed to validate this in the future.

## Data Availability

All the data generated or analyzed during the study are available and included in this published article.

## References

[1] Zhou Z, Hu D, Chen J (2004). An epidemiological survey of atrial fibrillation in China. Chin J of Intern Med.

[2] Chugh SS, Havmoeller R, Narayanan K (2014). Worldwide epidemiology of atrial fibrillation: a global burden of disease 2010 study. Circulation.

[3] Schnabel RB, Yin X, Gona P (2015). 50 year trends in atrial fibrillation prevalence, incidence, risk factors, and mortality in the Framingham heart study: a cohort study. Lancet.

[4] Chiang C, Okumura K, Zhang S (2017). 2017 consensus of the Asia Pacific heart rhythm society on stroke prevention in atrial fibrillation. J Arrhythm.

[5] Dai Y, Wang X, Cao L (2004). Expression of extracellular signal-regulated kinase and angiotensin-converting enzyme in human atria during atrial fibrillation. J Huazhong Univ Sci Technol.

[6] Soliman EZ, Safford MM, Muntner P (2014). Atrial fibrillation and the risk of myocardial infarction. JAMA Intern Med.

[7] Staerk L, Sherer JA, Ko D (2017). Atrial fibrillation: epidemiology, pathophysiology, and clinical outcomes”. Circ Res.

[8] Böhm M, Ezekowitz MD, Connolly SJ (2015). Changes in renal function in patients with atrial fibrillation: an analysis from the RE-LY trial. J Am Coll Cardiol.

[9] Zhang Y, Zhou S (2017). A review on treating atrial fibrillation by acupuncture. Clin J Chin Med.

[10] Liu C (2019). ‘Evidence-based medical proofs and future design thinking of acupuncture for treating cardiovascular diseases. Chin J Integr Tradit West Med.

[11] Liu F, Tan A, Peng C (2021). Efficacy and safety of scalp acupuncture for insomnia: a systematic review and meta-analysis. Evid-Based Complement Altern Med.

[12] Hindricks G, Potpara T, Dagres N (2021). 2020 ESC guidelines for the diagnosis and management of atrial fibrillation developed in collaboration with the European Association for Cardio-Thoracic Surgery (EACTS): the task force for the diagnosis and management of atrial fibrillation of the European Society Of Cardiology (ESC) developed with the special contribution of the European heart rhythm association (EHRA) of the ESC. Eur Heart J.

[13] Yin J, Yang M, Yu S (2019). Effect of acupuncture at Neiguan point combined with amiodarone therapy on early recurrence after pulmonary vein electrical isolation in patients with persistent atrial fibrillation. J Cardiovasc Electrophysiol.

[14] Xu B (2015). Effect of acupuncture combined with wenxinkeli on patients with paroxysmal atrial fibrillation. Med Recap.

[15] Xia Y, Fang G, Qiu X (2014). 50 cases of paroxysmal rapid atrial fibrillation treated with acupuncture. Chin Med Mod Distance Educ China.

[16] Yan Y, Li B, Wu Y (2014). Clinical observation on the combination of acupuncture and medicine in the treatment of paroxysmal atrial fibrillation. Zhejiang J Tradit Chin Med.

[17] Han B (2021). Clinical observation of 62 cases of paroxysmal rapid atrial fibrillation treated with acupuncture and drugs. J Emerg Tradit Chin Med.

[18] Li C, Chen F, Yang X (2012). Effectiveness and safety of atrial fibrillation treatment with buried threads at the Neiguan acupoint. J New Chin Med.

[19] Lomuscio A, Belletti S, Battezzati PM (2011). Efficacy of acupuncture in preventing atrial fibrillation recurrences after electrical cardioversion. J Cardiovasc Electrophysiol.

[20] Xu H, Zhang Y (2007). Comparison between therapeutic effects of acupuncture and intravenous injection of amiodarone in the treatment of paroxymal atrial fibrillation and atrial flutter. Zhongguo Zhen Jiu.

[21] Wang Y (2007). Clinical observation of 27 cases of idiopathic atrial fibrillation in the elderly treated with micro-needle knife. J Huaihai Med.

[22] Zhang Y, Du Q, Zhang Z (2003). Clinical study of 110 cases of tachyarrhythmias treated with acupuncture at Yingxiang point. Chin J Pract Med Res.

[23] Jiao Y, Gao S, Mu S (1997). Clinical efficacy of the application of ping fibrillation cream to treat atrial fibrillation. Chin J Tradit Med Science Technol.

[24] Andrade J, Khairy P, Dobrev D (2014). The clinical profile and pathophysiology of atrial fibrillation: relationships among clinical features, epidemiology, and mechanisms. Circ Res.

[25] Yao F, Wang Y (2010). Study on the effect of curing fast arrhythmia by acupuncture neiguan point of rabbit”. Inf Tradit Chin Med.

[26] Zhu P (2013). Study on the mechanism of acupuncture preconditioning treating big rats with paroxysmal atrial fibrillation.

[27] Liu H (2014). Effect of acupuncture preconditioning on QTC and atrial muscle ultrastructure of paroxysmal atrial fibrillation rats.

[28] Song Q (2014). The effect of acupuncture intervention on P-wave dispersion and atrial muscle ultrastructure in rats with paroxysmal atrial fibrillation.

[29] Lin R (2012). A study on the effect of needling Shenmen(HT7) on heart rate and heart rate variability.

[30] Liu L, Chen J, Wu Q (2010). Analysis of the mechanism by which acupuncture at Shen Men acupoint affects heart rate based on heart rate variability. J of Clin Acupunct Moxib.

[31] Luo L, Shen Y, Chen H (2009). Shanghai journal of acupuncture and moxibustion. Shanghai J Acupunct Moxib.

[32] Gong Z, Yan Z, Liu Q (2021). Effect of intranasal acupuncture on neurogenic inflammation in allergic rhinitis rabbits. Acupunct Res.

[33] Yang S, Wu J, Zhang Q (2018). Catgut implantation at acupoint reduces immune reaction in a rat model of allergic rhinitis. Evid-based complement altern med.

[34] Lomuscio A, Belletti S, Battezzati PM (2011). Efficacy of acupuncture in preventing atrial fibrillation recurrences after electrical cardioversion. J Cardiovasc Electrophysiol.

[35] Yu J, Meng Q, Zhang Y (2013). Double-way regulation effect of electroacupuncture at Neiguan point on cardiac arrhythmia rats and discussion on its central mechanism. J of Clin Acupunct Moxib.

[36] Li L, Huang J, Wang G (2020). Efficacy observation of acupuncture combined with Gegen Guizhi Gancao decoction for arrhythmias. Shanghai J Acupunct Moxib..

[37] Guo Y, Lip GYH, Apostolakis S (2012). Inflammation in atrial fibrillation. J Am Coll Cardiol.

[38] Lin L, Wang L, Yang J (2019). Researches status on time-effect of acupuncture. Chin Acupunct Moxib.

[39] Lee M, Lee S, Kim E (2018). Evaluation of bleeding-related adverse events following acupuncture treatment in patients on anticoagulant or antiplatelet drugs: a prospective observational study. Complement Ther Med.

[40] Kwon S, Jung W, Yang S (2018). Safety of acupuncture in patients taking newer oral anticoagulants: a retrospective chart review study. Evid-based complement altern med.

[41] Mcculloch M, Nachat A, Schwartz J (2015). Acupuncture safety in patients receiving anticoagulants: a systematic review. Perm J.

[42] Kuck KH (2016). Cryoballoon or radiofrequency ablation for paroxysmal atrial fibrillation. New Engl J Med..

[43] Wilber DJ (2010). Comparison of antiarrhythmic drug therapy and radiofrequency catheter ablation in patients with paroxysmal atrial fibrillation: a randomized controlled trial. JAMA.

[44] Senatore G (2005). Role of transtelephonic electrocardiographic monitoring in detecting short-term arrhythmia recurrences after radiofrequency ablation in patients with atrial fibrillation. J Am Coll Cardiol.

